# A Seed-Based Plant Propagation Algorithm: The Feeding Station Model

**DOI:** 10.1155/2015/904364

**Published:** 2015-03-02

**Authors:** Muhammad Sulaiman, Abdellah Salhi

**Affiliations:** ^1^Department of Mathematical Sciences, University of Essex, Colchester CO4 3SQ, UK; ^2^Department of Mathematics, Abdul Wali Khan University, Mardan, Khyber Pakhtunkhwa, Pakistan

## Abstract

The seasonal production of fruit and seeds is akin to opening a feeding station, such as a restaurant. Agents coming to feed on the fruit are like customers attending the restaurant; they arrive at a certain rate and get served at a certain rate following some appropriate processes. The same applies to birds and animals visiting and feeding on ripe fruit produced by plants such as the strawberry plant. This phenomenon underpins the seed dispersion of the plants. Modelling it as a queuing process results in a seed-based search/optimisation algorithm. This variant of the Plant Propagation Algorithm is described, analysed, tested on nontrivial problems, and compared with well established algorithms. The results are included.

## 1. Introduction

Plants have evolved a variety of ways to propagate. Propagation with seeds is perhaps the most common of them all and one which takes advantage of all sorts of agents ranging from wind to water, birds, and animals. In [1] a Plant Propagation Algorithm based on the way the strawberry plant propagates using runners has been introduced. Here, we consider the case where the strawberry plant uses seeds to propagate.

**Figure 1:**
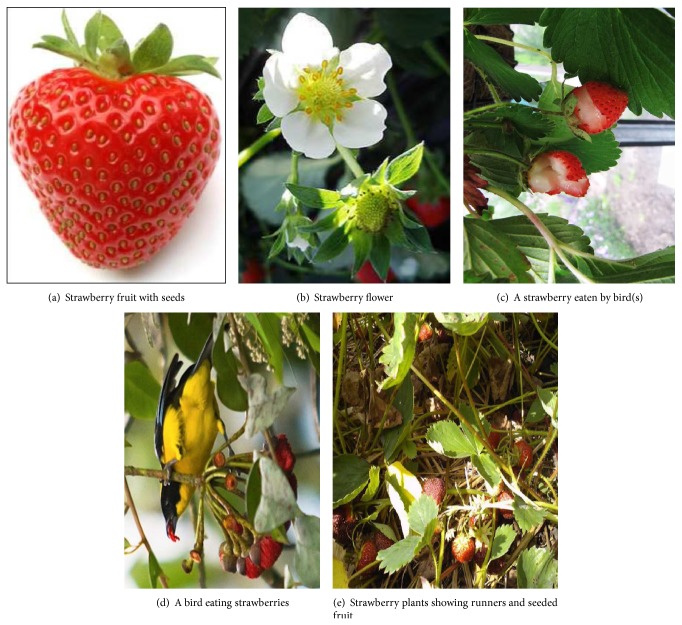
Strawberry plant propagation: through seed dispersion [25–28].

Plants rely heavily on the dispersion of their seeds to colonise new territories and to improve their survival [2, 3]. There are a lot of studies and models of seed dispersion particularly for trees [2–6]. Dispersion by wind and ballistic means is probably the most studied of all approaches [7–9]. However, in the case of the strawberry plant, given the way the seeds stick to the surface of the fruit (Figure 1(a)) [10], dispersion by wind or mechanical means is very limited. Animals, however, and birds in particular are the ideal agents for dispersion [2, 3, 11, 12] in this case.

There are many biologically inspired optimization algorithms in the literature [13, 14]. The Flower Pollination Algorithm (FPA) is inspired by the pollination of flowers through different agents [8]; the swarm data clustering algorithm is inspired by pollination by bees [15]; Particle Swarm Optimization (PSO) is inspired by the foraging behavior of groups of animals and insects [16, 17]; the Artificial Bee Colony (ABC) simulates the foraging behavior of honey bees [18, 19]; the Firefly algorithm is inspired by the flashing fireflies when trying to attract a mate [20, 21]; the Social Spider Optimization (SSO) algorithm is inspired by the cooperative behavior of social spiders [22]. The list could easily be extended.

The Plant Propagation Algorithm (PPA) also known as the strawberry algorithm was inspired by the way plants and specifically the strawberry plants propagate using runners [1, 23]. The attraction of PPA is that it can be implemented easily for all sorts of optimization problems. Moreover, it has few algorithm specific arbitrary parameters. It follows the principle that plants in good spots with plenty of nutrients will send many short runners. They send few long runners when in nutrient poor spots. With long runners PPA tries to explore the search space while short runners enable it to exploit the solution space well. In this paper, we investigate an alternative PPA which is entirely based on the propagation by seeds of the strawberry plant. Because of the periodic nature of fruit and seed production, it amounts to setting up a feeding station for the attention of potential seed-dispersing agents [24], Hence the feeding station model used here and the resulting Seed-Based Plant Propagation Algorithm or SbPPA.

SbPPA is tested on both unconstrained and constrained benchmark problems also used in [22, 29, 30]. Experimental results are presented in Tables 4–7 in terms of best, mean, worst, and standard deviation for all algorithms. The paper is organised as follows. In Section 2 we briefly introduce the feeding station model representing strawberry plants in fruit and the main characteristics of the paths followed by different agents that disperse the seeds.  Section 3 presents the SbPPA in pseudocode form. The experimental settings, results, and convergence graphs for different problems are given in Section 4.

## 2. Aspects of the Feeding Station Model

Some animals and plants depend on each other to conserve their species [31]. Thus, many plants require, for effective seed dispersal, the visits of frugivorous birds or animals according to a certain distribution [2, 3, 32, 33].

Seed dispersal by different agents is also called “seed shadow” [32]; this shows the abundance of seeds spread globally or locally around parent plants. Here a queuing model is used which, in the context of a strawberry feeding station model, involves two parts:

1. the quantity of fruit or seeds available to agents which implies the rate at which the agents will visit the plants,
2. a probability density function that tells us about the service rate with which the agents are served by the plants.

The model estimates the quantity of seeds that is spread locally compared to that dispersed globally [34–38]. There are two aspects that need to be balanced: exploitation, which is represented by the dispersal of seeds around the plants, and exploration which ensures that the search space is well covered.

Agents arrive at plants in a random process. Assume that at most one agent arrives to the plants in any unit of time (orderliness condition). It is further supposed that the probability of arrivals of agents to the plants remains the same for a particular period of time. This period corresponds to when the plants are in fruit and during which time the number of visitors is stable (stationarity condition). Furthermore, it is assumed that the arrival of one agent does not affect the rest of arrivals (independence).

With these assumptions in mind, the arrival of agents to plants follows a Poisson process [39, 40], which can be formally described as follows. Let *X*′ be the random variable representing the number of arrivals per unit of time *t*. Then, the probability of *k* arrivals over *t* is

(1) $$\begin{array}{c} P\left(X^{\prime }=k\right)=\frac{{\left(\lambda t\right)}^{k}e^{-\lambda t}}{k!}, \end{array}$$

where *λ* denotes the mean arrival rate of agents per time unit *t*. On the other hand, the time taken by agents in successfully eating fruit and leaving to disperse its seeds, in other words the service time for agents, is expressed by a random variable which follows the exponential probability distribution [41]. This can be expressed as follows:

(2) $$\begin{array}{c} S\left(t\right)=\mu e^{-\mu t}, \end{array}$$

where *μ* is the average number of agents that can feed at time *t*. Let us assume that the arrival rate of agents is less than the fruits available on all plants per unit of time; therefore *λ* < *μ*.

We assume that the system is in steady state. Let *A* denote the average number of agents in the strawberry field (some already eating and the rest waiting to feed) and *A*_*q*_ the average number of agents waiting to get the chance to feed. If we denote the average number of agents eating fruits by *λ*/*μ*, then by Little's formula [42], we have

(3) $$\begin{array}{c} A=A_{q}+\frac{\lambda }{\mu }. \end{array}$$

Since the plant needs to maximise dispersion, this is equivalent to having a large *A*_*q*_ in (3). Therefore, from this equation, we need to solve the following problem:

(4) Maximize Aq=A−λμ,subject  to g1(λ,μ)=λ<μ+1,hhhhhhihhhhIhhhλ>0, μ>0,

where *A* = 10, which represents the population size in the implementation. The simple limits on the variables are 0 < *λ*, *μ* ≤ 100. The optimum solution to this particular problem is *λ* = 1.1, *μ* = 0.1, and *A*_*q*_ = 1.

Frugivores may travel far away from the plants and hence will disperse the seeds far and wide. This feeding behaviour typically follows a Lévy distribution [43–45]. In the following we present some basic facts about it.

### 2.1. Lévy Distribution

The Lévy distribution is a probability density distribution for random variables. Here the random variables represent the directions of flights of arbitrary birds. This function ranges over real numbers in the domain represented by the problem search space.

The flight lengths of the agents served by the plants follow a heavy tailed power law distribution [14], represented by

(5) $$\begin{array}{c} L\left(s\right)\sim {\left|s\right|}^{-1-\beta }, \end{array}$$

where *L*(*s*) denotes the Lévy distribution with index *β* ∈ (0,2). Lévy flights are unique arbitrary excursions whose step lengths are drawn from (5). An alternative form of Lévy distribution is [14]

(6) L(s,γ,μ)=γ2π1s−μ3/2 ·exp⁡−γ2s−μ,0<μ<s<∞,0,Otherwise.

This implies that

(7) $$\begin{array}{c} \underset{s\to \infty }{\lim}L\left(s,\gamma ,\mu \right)\approx \sqrt{\frac{\gamma }{2\pi }}{\left(\frac{1}{s}\right)}^{3/2}. \end{array}$$

In terms of the Fourier transform [14], the limiting value of *L*(*s*) can be written as

(8) $$\begin{array}{c} \underset{s\to \infty }{\lim}L\left(s\right)=\frac{\alpha \beta \Gamma \left(\beta \right)\sin\left(\pi \beta /2\right)}{\pi {\left|s\right|}^{1+\beta }}, \end{array}$$

where Γ(*β*) is the Gamma function [46], defined by

(9) $$\begin{array}{c} \Gamma \left(\beta \right)=\overset{\infty }{\underset{0}{\int }}x^{\beta -1}e^{-x}dx. \end{array}$$

The steps *L*(*s*) are generated by Mantegna's algorithm [14]. This algorithm ensures that the behaviour of Lévy flights is symmetric and stable as shown in Figure 3(b).

## 3. Strawberry Plant Propagation Algorithm: The Feeding Station Model

**Figure 2:**
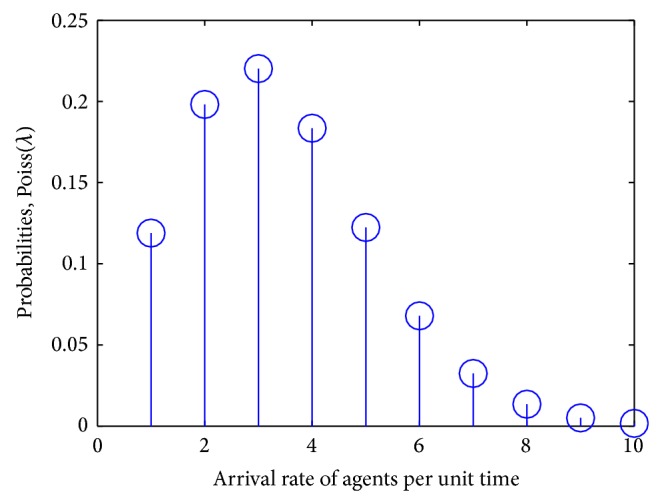
Distribution of agents arriving at strawberry plants to eat fruit and disperse seeds.

We assume that the arrival of different agents (birds and animals) to the plants to feed is according to the Poisson distribution [40]. As per the solution of problem (4), the mean arrival rate is *λ* = 1.1, and NP = 10 is the size of the agents population. Let *k* = 1,2,…, *A* be the possible numbers of agents visiting the plants per unit time. With these assumptions the graphic representation of (1) results in Figure 2.

As already stated, it is essential in this algorithm to balance exploration and exploitation. To this end, we choose a threshold value of the Poisson probability that dictates how much exploration and exploitation are done during the search. The probability Poiss(*λ*) < 0.05 means that exploitation is covered. In this case, (10) below is used, which helps the algorithm to search locally:

(10) xi,j∗=xi,j+ξjxi,j−xl,jif  PR≤0.8;  j=1,2,…,n; iii,l=1,2,…,NP;  i≠l,xi,j,Otherwise,

where PR denotes the rate of dispersion of the seeds locally, around SP; *x*_*i*,j_^*^  and  *x*_*i*,*j*_ ∈ [*a*_*j*_ *b*_*j*_] are the *j*th coordinates of the seeds *X*_i_^*^ and *X*_*i*_, respectively; *a*_*j*_ and *b*_*j*_ are the *j*th lower and upper bounds defining the search space of the problem and *ξ*_*j*_ ∈ [−1   1]. The indices *l*  and  *i* are mutually exclusive.

**Figure 3:**
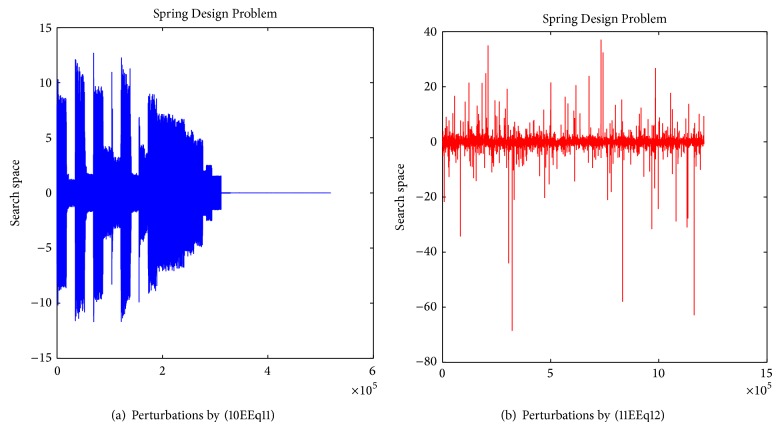
Overall performance of SbPPA on Spring Design Problem.

On the other hand, if Poiss(*λ*) ≥ 0.05 then global dispersion of seeds becomes more prominent. This is implemented by using the following equation:

(11) xi,j∗=xi,j+Lixi,j−θjif  PR≤0.8,  θj∈ajbj,hhi=1,2,…,NP; hhj=1,2,…,n,xi,j,Otherwise,

where *L*_*i*_ is a step drawn from the Lévy distribution [14] and *θ*_*j*_ is a random coordinate within the search space. Equations (10) and (11) perturb the current solution, the results of which can be seen in Figures 3(a) and 3(b), respectively.

**Figure 4:**
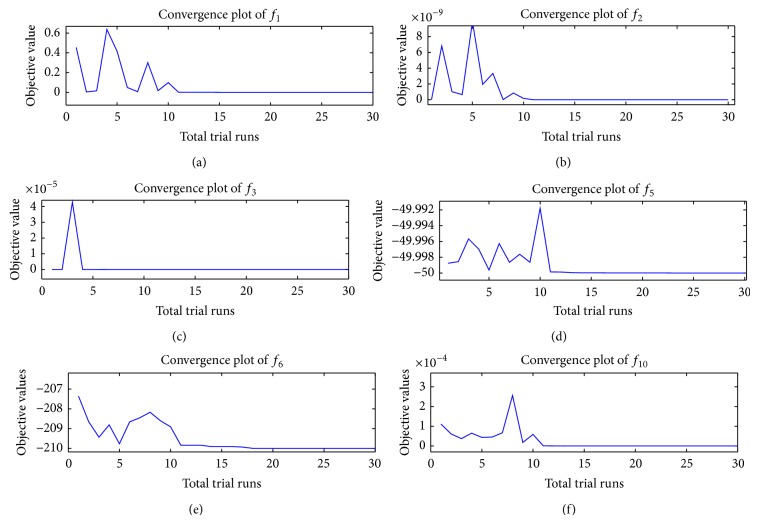
Performance of SbPPA on unconstrained global optimization problems.

**Figure 5:**
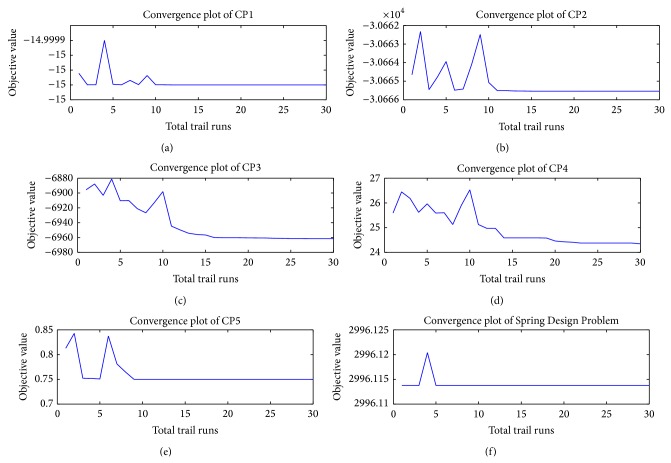
Performance of SbPPA on constrained global optimization problems (see Appendices).

As mentioned in Algorithm 1, we first collect the best solutions from the first NP trial runs to form a population of potentially good solutions denoted by pop_best_. The convergence rate of SbPPA is shown in Figures 4 and 5 for different test problems used in our experiments (see Appendices). The statistics values best, worst, mean, and standard deviation are calculated based on pop_best_.

The seed-based propagation process of SP can be represented in the following steps.

1. The dispersal of seeds in the neighbourhood of the SP, as shown in Figure 1(e), is carried out either by fruits fallen from strawberry plants after they become ripe or by agents. The step lengths for this phase are calculated using (10).
2. Seeds are spread globally through agents, as shown in Figures 1(c) and 1(d). The step lengths for these travelling agents are drawn from the Lévy distribution [14].
3. The probabilities, Poiss(*λ*), that a certain number *k* of agents will arrive to SP to eat fruits and disperse it, is used as a balancing factor between exploration and exploitation.

For implementation purposes, we assume that each SP produces one fruit, and each fruit is assumed to have one seed; by a solution *X*_*i*_ we mean the current position of the *i*th seed to be dispersed. The number of seeds in the population is denoted by NP. Initially we generate a random population of NP seeds using

(12) $$\begin{array}{c} x_{i,j}=a_{j}+\left(b_{j}-a_{j}\right)\eta_{j},\;j=1,\ldots ,n, \end{array}$$

where *x*_*i*,*j*_ ∈ [*a*_*j*_ *b*_*j*_] is the *j*th coordinate of solution *X*_*i*_, *a*_*j*_ and *b*_*j*_ are the *j*th coordinates of the bounds describing the search space of the problem, and *η*_*j*_ ∈ (0   1). This means that *X*_*i*_ = [*x*_*i*,*j*_], for  *j* = 1,…, *n*, represents the position of the *j*th seed in population pop.

## 4. Experimental Settings and Discussion

In our experiments we tested SbPPA against some recently developed algorithms and some well established and standard ones. Our set of test problems includes benchmark constrained and unconstrained optimization problems [22, 30, 48, 49]. The results are compared in terms of statistics (best, worst, mean and standard deviation) for solutions obtained by SbPPA; ABC [18, 50]; PSO [51]; FF [21]; HPA [29]; SSO-C [22]; Classical Evolutionary Programming (CEP) [30]; and Fast Evolutionary Programming (FEP) [30]. The detailed descriptions of these problems are given in Appendices.

In Tables 4 and 7, the significance of results is shown in terms of win/tie/loss (see Table  2 in [52]) according to the following notations:

- (+) when SbPPA is better;
- (≈) when the results are approximately the same as those obtained with SbPPA;
- (−) when SbPPA is worse.

Moreover, in Tables 5 and 6 the significance of results obtained with SbPPA is highlighted.

### 4.1. Parameter Settings

The parameter settings are given in Tables 1–3.

## 5. Conclusion

In this paper, a new metaheuristic referred to as the Seed-Based Plant Propagation Algorithm (SbPPA) [47] has been proposed. Plants have evolved a variety of ways to propagate. Propagation through seeds is perhaps the most common of them all and one which takes advantage of all sorts of agents ranging from wind to water, birds, and animals. The strawberry plant uses both runners and seeds to propagate. Here we consider the propagation through seeds that the strawberry plant has evolved, to design an efficient optimization algorithm.

To capture the dispersal process, we adopt a queuing approach which, given the extent of fruit produced, indicates the extent of seeds dispersed and hence the effectiveness of the search/optimization algorithm based on this process. Looking at the random process of agents using the plants (feeding station) it is reasonable to assume that it is of the Poisson type. On the other hand, the time taken by agents in successfully eating fruit and leaving to disperse its seeds, in other words the service time for agents, is expressed by a random variable which follows the exponential probability distribution. To this end, we choose a threshold value of the Poisson probability that dictates how much exploration and exploitation are done during the search. An alternative strategy has been adopted here. This strategy consists in making sure that the initial population is as good as the user can afford it to be by using best solutions found so far. The effects of this strategy on convergence are shown through convergence plots of Figures 4 and 5, for some of the solved problems. SbPPA is easy to implement as it requires less arbitrary parameter settings than other algorithms. The success rate of SbPPA increases as it gets its population of best solutions. It has been implemented for both unconstrained and constrained optimization problems. Its performance, compared to that of other algorithms, points to SbPPA as being superior.

## Figures and Tables

**Algorithm 1:**
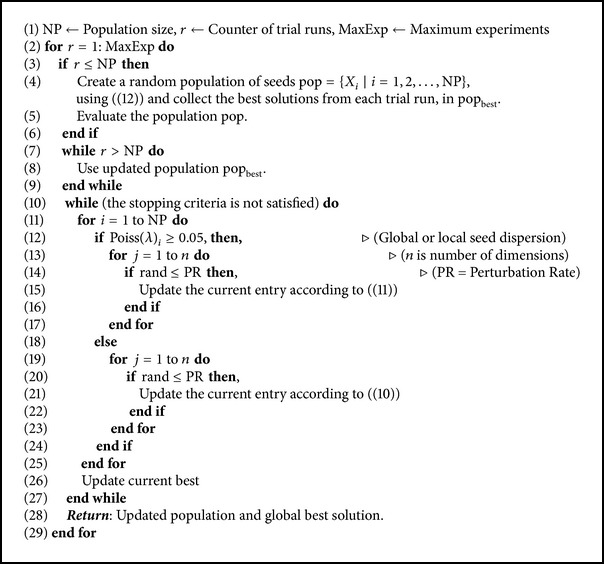
Seed-based Plant Propagation Algorithm (SbPPA) [47].

**Table 1:** Parameters used for each algorithm for solving unconstrained global optimization problems *f*_1_–*f*_10_. All experiments are repeated 30 times.

PSO [16, 29]	ABC [18, 29]	HPA [29]	SbPPA [47]
*M* = 100	SN = 100	Agents = 100	NP = 10
$G_{\max}=\frac{\left(\text{Dimension}\times 20,000\right)}{M}$	MCN = $\frac{\left(\text{Dimension}\times 20,000\right)}{\text{SN}}$	Iteration number = $\frac{\left(\text{Dimension}\times 20,000\right)}{\text{Agents}}$	Iteration number = $\frac{\left(\text{Dimension}\times 20,000\right)}{\text{NP}}$
*c*_1_ = 2	MR = 0.8	*c*_1_ = 2	PR = 0.8
*c*_2_ = 2	Limit = $\frac{\left(\text{SN}\times \text{dimension}\right)}{2}$	*c*_2_ = 2	Poiss(λ) = 0.05
*W* = $\frac{\left(G_{\max}-\text{iteratio}{\text{n}}_{\text{index}}\right)}{G_{\max}}$	—	Limit = $\frac{\left(\text{Agents}\times \text{dimension}\right)}{2}$	—
—	—	*W* = $\frac{\left(\text{Iteration}\;\;\text{number}-\text{iteratio}{\text{n}}_{\text{index}}\right)}{\text{Iteration}\;\;\text{number}}$	—

**Table 2:** Experimental setup used for each algorithm for solving unconstrained global optimization problems *f*_11_–*f*_26_. All experiments are repeated 50 times.

CEP [30, 53, 54]	FEP [30]	SbPPA
Population size *μ* = 100	Population size μ = 100	NP = 10
Tournament size *q* = 10	Tournament size *q* = 10	PR = 0.8
η = 3.0	η = 3.0	Poiss(*λ*) = 0.05

**Table 3:** Parameters used for each algorithm for solving constrained optimization problems. All experiments are repeated 30 times.

PSO [51]	ABC [50]	FF [21]	SSO-C [22]	SbPPA [47]
*M* = 250	SN = 40	Fireflies = 25	*N* = 50	NP = 10
*G*_max⁡_ = 300	MCN = 6000	Iteration number = 2000	Iteration number = 500	Iteration number = 800
*c*_1_ = 2	MR = 0.8	*q* = 1.5	PF = 0.7	PR = 0.8
*c*_2_ = 2	—	α = 0.001	—	Poiss(λ) = 0.05
Weight factors = 0.9 to 0.4	—	—	—	—

**Table 4:** Results obtained by SbPPA, HPA, PSO, and ABC. All problems in this table are unconstrained.

Fun.	Dim	Algorithm	Best	Worst	Mean	SD
*f*_1_	4	ABC	(+) 0.0129	(+) 0.6106	(+) 0.1157	(+) 0.111
		PSO	(−) 6.8991*E* − 08	(+) 0.0045	(+) 0.001	(+) 0.0013
		HPA	(+) 2.0323*E* − 06	(+) 0.0456	(+) 0.009	(+) 0.0122
		SbPPA	1.08*E* − 07	7.05*E* − 06	3.05*E* − 06	3.14*E* − 06

*f*_2_	2	ABC	(+) 1.2452*E* − 08	(+) 8.4415*E* − 06	(+) 1.8978*E* − 06	(+) 1.8537*E* − 06
		PSO	(≈) 0	(≈) 0	(≈) 0	(≈) 0
		HPA	(≈) 0	(≈) 0	(≈) 0	(≈) 0
		SbPPA	0	0	0	0

*f*_3_	2	ABC	(≈) 0	(+) 4.8555*E* − 06	(+) 4.1307*E* − 07	(+) 1.2260*E* − 06
		PSO	(≈) 0	(+) 3.5733*E* − 07	(+) 1.1911*E* − 08	(+) 6.4142*E* − 08
		HPA	(≈) 0	(≈) 0	(≈) 0	(≈) 0
		SbPPA	0	0	0	0

*f*_4_	2	ABC	(≈) − 1.03163	(≈) − 1.03163	(≈) − 1.03163	(≈) 0
		PSO	(≈) − 1.03163	(≈) − 1.03163	(≈) − 1.03163	(≈) 0
		HPA	(≈) − 1.03163	(≈) − 1.03163	(≈) − 1.03163	(≈) 0
		SbPPA	−1.031628	−1.031628	−1.031628	0

*f*_5_	6	ABC	(≈) − 50.0000	(≈) − 50.0000	(≈) − 50.0000	(−) 0
		PSO	(≈) − 50.0000	(≈) − 50.0000	(≈) − 50.0000	(−) 0
		HPA	(≈) − 50.0000	(≈) − 50.0000	(≈) − 50.0000	(−) 0
		SbPPA	− 50.0000	− 50.0000	− 50.0000	5.88*E* − 09

*f*_6_	10	ABC	(+) − 209.9929	(+) − 209.8437	(+) − 209.9471	(+) 0.044
		PSO	(≈) − 210.0000	(≈) − 210.0000	(≈) − 210.0000	(−) 0
		HPA	(≈) − 210.0000	(≈) − 210.0000	(≈) − 210.0000	(+) 1
		SbPPA	− 210.0000	− 210.0000	− 210.0000	4.86*E* − 06

*f*_7_	30	ABC	(+) 2.6055*E* − 16	(+) 5.5392*E* − 16	(+) 4.7403*E* − 16	(+) 9.2969*E* − 17
		PSO	(≈) 0	(≈) 0	(≈) 0	(≈) 0
		HPA	(≈) 0	(≈) 0	(≈) 0	(≈) 0
		SbPPA	0	0	0	0

*f*_8_	30	ABC	(+) 2.9407*E* − 16	(+) 5.5463*E* − 16	(+) 4.8909*E* − 16	(+) 9.0442*E* − 17
		PSO	(≈) 0	(≈) 0	(≈) 0	(≈) 0
		HPA	(≈) 0	(≈) 0	(≈) 0	(≈) 0
		SbPPA	0	0	0	0

*f*_9_	30	ABC	(≈) 0	(+) 1.1102*E* − 16	(+) 9.2519*E* − 17	(+) 4.1376*E* − 17
		PSO	(≈) 0	(+) 1.1765*E* − 01	(+) 2.0633*E* − 02	(+) 2.3206*E* − 02
		HPA	(≈) 0	(≈) 0	(≈) 0	(≈) 0
		SbPPA	0	0	0	0

*f*_10_	30	ABC	(+) 2.9310*E* − 14	(+) 3.9968*E* − 14	(+) 3.2744*E* − 14	(+) 2.5094*E* − 15
		PSO	(≈) 7.9936*E* − 15	(+) 1.5099*E* − 14	(+) 8.5857*E* − 15	(+) 1.8536*E* − 15
		HPA	(≈) 7.9936*E* − 15	(+) 1.5099*E* − 14	(+) 1.1309*E* − 14	(+) 3.54*E* − 15
		SbPPA	7.994*E* − 15	7.99361*E* − 15	7.994*E* − 15	7.99361*E* − 15

**Table 5:** Results obtained by SbPPA, CEP, and FEP. All problems in this table are unconstrained [30].

Function number	Algorithm	Maximum generations	Mean	SD
*f*_11_	CEP	2000	2.60*E* − 03	1.70*E* − 04
	FEP		8.10*E* − 03	7.70*E* − 04
	SbPPA		9.45**E** − 13	4.08**E** − 12

*f*_12_	CEP	5000	2	1.2
	FEP		0.3	0.5
	SbPPA		3.93**E** − 02	3.76**E** − 02

*f*_13_	CEP	20000	6.17	13.61
	FEP		5.06	5.87
	SbPPA		1.86*E* + 01	2.25*E* + 00

*f*_14_	CEP	1500	577.76	1125.76
	FEP		0	0
	SbPPA		0	0

*f*_15_	CEP	3000	1.80*E* − 02	6.40*E* − 03
	FEP		7.60*E* − 03	2.60*E* − 03
	SbPPA		3.61**E** − 03	1.31**E** − 03

*f*_16_	CEP	9000	− 7.92*E* + 03	6.35*E* + 02
	FEP		− 1.26*E* + 04	5.26*E* + 01
	SbPPA		− 1.16*E* + 04	6.04*E* + 01

*f*_17_	CEP	5000	89	23.1
	FEP		4.60*E* − 02	1.20*E* − 02
	SbPPA		8.73**E** + 00	9.88**E** − 01

*f*_18_	CEP	100	0.398	1.50*E* − 07
	FEP		0.398	1.50*E* − 07
	SbPPA		3.98*E* − 01	0

**Table 6:** Results obtained by SbPPA, CEP, and FEP. All problems in this table are unconstrained [30].

Function number	Algorithm	Maximum generations	Mean	SD
*f*_19_	CEP	100	3	0
	FEP		3.02	0.11
	SbPPA		3	3.05**E** − 15

*f*_20_	CEP	100	− 3.86*E* + 00	1.40*E* − 02
	FEP		− 3.86*E* + 00	1.40*E* − 05
	SbPPA		− 3.86*E* + 00	2.75**E** − 15

*f*_21_	CEP	200	− 3.28*E* + 00	5.80*E* − 02
	FEP		− 3.27*E* + 00	5.90*E* − 02
	SbPPA		− 3.32**E** + 00	2.91**E** − 14

*f*_22_	CEP	100	− 6.86*E* + 00	2.67*E* + 00
	FEP		− 5.52*E* + 00	1.59*E* + 00
	SbPPA		− 1.02**E** + 01	4.30**E** − 09

*f*_23_	CEP	100	− 8.27	2.95
	FEP		− 5.52	2.12
	SbPPA		− 1.04**E** + 01	7.73**E** − 09

*f*_24_	CEP	100	− 9.1	2.92
	FEP		− 6.57	3.14
	SbPPA		− 1.05**E** + 01	1.03**E** − 07

*f*_25_	CEP	100	1.66	1.19
	FEP		1.22	0.56
	SbPPA		9.98**E** − 01	1.13**E** − 16

*f*_26_	CEP	4000	4.70*E* − 04	3.00*E* − 04
	FEP		5.00*E* − 04	3.20*E* − 04
	SbPPA		3.07**E** − 04	6.80**E** − 15

**Table 7:** Results obtained by SbPPA, PSO, ABC, FF, and SSO-C. All problems in this table are standard constrained optimization problems.

Fun. name	Optimal	Algorithm	Best	Mean	Worst	SD
CP1	−15	PSO	(≈) − 15	(≈) − 15	(≈) − 15	(−) 0
		ABC	(≈) − 15	(≈) − 15	(≈) − 15	(−) 0
		FF	(+) 14.999	(+) 14.988	(+) 14.798	(+) 6.40*E* − 07
		SSO-C	(≈) − 15	(≈) − 15	(≈) − 15	(−) 0
		SbPPA	− 15	− 15	− 15	1.95*E* − 15

CP2	− 30665.539	PSO	(≈) − 30665.5	(+) − 30662.8	(+) − 30650.4	(+) 5.20*E* − 02
		ABC	(≈) − 30665.5	(+) − 30664.9	(+) − 30659.1	(+) 8.20*E* − 02
		FF	(≈) − 3.07*E* + 04	(+) − 30662	(+) − 30649	(+) 5.20*E* − 02
		SSO-C	(≈) − 3.07*E* + 04	(≈) − 30665.5	(+) − 30665.1	(+) 1.10*E* − 04
		SbPPA	− 30665.5	− 30665.5	− 30665.5	2.21*E* − 06

CP3	− 6961.814	PSO	(+) − 6.96*E* + 03	(+) − 6958.37	(+) − 6942.09	(+) 6.70*E* − 02
		ABC	(−) − 6961.81	(+) − 6958.02	(+) − 6955.34	(−) 2.10*E* − 02
		FF	(+) − 6959.99	(+) − 6.95*E* + 03	(+) − 6947.63	(−) 3.80*E* − 02
		SSO-C	(−) − 6961.81	(+) − 6961.01	(+) − 6960.92	(−) 1.10*E* − 03
		SbPPA	− 6961.5	− 6961.38	− 6961.45	0.043637

CP4	24.306	PSO	(−) 24.327	(+) 2.45*E* + 01	(+) 24.843	(+) 1.32*E* − 01
		ABC	(+) 24.48	(+) 2.66*E* + 01	(+) 28.4	(+) 1.14
		FF	(−) 23.97	(+) 28.54	(+) 30.14	(+) 2.25
		SSO-C	(−) 24.306	(−) 24.306	(−) 24.306	(−) 4.95*E* − 05
		SbPPA	24.34442	24.37536	24.37021	0.012632

CP5	− 0.7499	PSO	(≈) − 0.7499	(+) − 0.749	(+) − 0.7486	(+) 1.20*E* − 03
		ABC	(≈) − 0.7499	(+) − 0.7495	(+) − 0.749	(+) 1.67*E* − 03
		FF	(+) − 0.7497	(+) − 0.7491	(+) − 0.7479	(+) 1.50*E* − 03
		SSO-C	(≈) − 0.7499	(≈) − 0.7499	(≈) − 0.7499	(−) 4.10*E* − 09
		SbPPA	0.7499	0.749901	0.7499	1.66*E* − 07

Spring Design Problem	Not known	PSO	(+) 0.012858	(+) 0.014863	(+) 0.019145	(+) 0.001262
		ABC	(≈) 0.012665	(+) 0.012851	(+) 0.01321	(+) 0.000118
		FF	(≈) 0.012665	(+) 0.012931	(+) 0.01342	(+) 0.001454
		SSO-C	(≈) 0.012665	(+) 0.012765	(+) 0.012868	(+) 9.29*E* − 05
		SbPPA	0.012665	0.012666	0.012666	3.39*E* − 10

Welded beam design problem	Not known	PSO	(+) 1.846408	(+) 2.011146	(+) 2.237389	(+) 0.108513
		ABC	(+) 1.798173	(+) 2.167358	(+) 2.887044	(+) 0.254266
		FF	(+) 1.724854	(+) 2.197401	(+) 2.931001	(+) 0.195264
		SSO-C	(≈) 1.724852	(+) 1.746462	(+) 1.799332	(+) 0.02573
		SbPPA	1.724852	1.724852	1.724852	4.06*E* − 08

Speed reducer design optimization	Not known	PSO	(+) 3044.453	(+) 3079.262	(+) 3177.515	(+) 26.21731
		ABC	(+) 2996.116	(+) 2998.063	(+) 3002.756	(+) 6.354562
		FF	(+) 2996.947	(+) 3000.005	(+) 3005.836	(+) 8.356535
		SSO-C	(≈) 2996.113	(≈) 2996.113	(≈) 2996.113	(+) 1.34*E* − 12
		SbPPA	2996.114	2996.114	2996.114	0

**Table 8:** Unconstrained global optimization problems (Set-1) used in our experiments.

Fun.	Fun. name	*D*	*C*	Range	Min	Formulation
*f*_1_	Colville	4	UN	[−10 10]^*D*^	0	*f*(*x*) = 100(*x*_1_^2^ − *x*_2_) + (*x*_1_ − 1)^2^ + (*x*_3_ − 1)^2^ + 90(*x*_3_^2^ − *x*_4_)^2^
						+10.1((*x*_2_ − 1)^2^ + (*x*_4_ − 1)^2^) + 19.8(*x*_2_ − 1)(*x*_4_ − 1)

*f*_2_	Matyas	2	UN	[−10 10]^*D*^	0	*f*(*x*) = 0.26(*x*_1_^2^ + *x*_2_^2^) − 0.48*x*_1_ *x*_2_

*f*_3_	Schaffer	2	MN	[−100 100]^*D*^	0	$f(x)=0.5+\frac{\sin^{2}\left(\sqrt{\sum_{i=1}^{n}‍x_{i}^{2}}\right)-0.5}{\left(1+0.001\left(\sum_{i=1}^{n}‍x_{i}^{2}\right)\right){}^{2}}$

*f*_4_	Six Hump Camel Back	2	MN	[−5 5]^*D*^	− 1.03163	$f(x)=4x_{1}^{2}-2.1x_{1}^{4}+\frac{1}{3}x_{1}^{6}+x_{1}x_{2}-4x_{2}^{2}+4x_{2}^{4}$

*f*_5_	Trid6	6	UN	[−36 36]^*D*^	− 50	*f*(*x*) = ∑_*i*=1_^6^‍(*x*_*i*_ − 1)^2^ − ∑_*i*=2_^6^‍*x*_*i*_ *x*_*i*−1_

*f*_6_	Trid10	10	UN	[−100 100]^*D*^	− 210	*f*(*x*) = ∑_*i*=1_^10^‍(*x*_*i*_ − 1)^2^ − ∑_*i*=2_^10^‍*x*_*i*_ *x*_*i*−1_

*f*_7_	Sphere	30	US	[−100 100]^*D*^	0	*f*(*x*) = ∑_*i*=1_^*n*^‍*x*_*i*_^2^

*f*_8_	SumSquares	30	US	[−10 10]^*D*^	0	*f*(*x*) = ∑_*i*=1_^*n*^‍*ix*_*i*_^2^

*f*_9_	Griewank	30	MN	[−600 600]^*D*^	0	$f(x)=\frac{1}{4000}\sum_{i=1}^{n}‍x_{i}^{2}-\prod_{i=1}^{n}\cos\left(\frac{x_{i}}{\sqrt{i}}\right)+1$

*f*_10_	Ackley	30	MN	[−32 32]^*D*^	0	$f(x)=-20\exp\left(-0.2\sqrt{\frac{1}{n}\sum_{i=1}^{n}‍x_{i}^{2}}\right)-\exp\left(\frac{1}{n}\sum_{i=1}^{n}‍\cos\left(2\pi x_{i}\right)\right)+20+e$

**Table 9:** Unconstrained global optimization problems (Set-2) used in our experiments [30].

Fun. number	Range	*D*	Function	Formulation	*f*_min⁡_

*f*_11_	[−10, 10]^*D*^	30	Schwefel's Problem 2.22	$f\left(x\right)=\sum_{i=1}^{n}‍|x_{i}|+\prod_{i=1}^{n}\left|x_{i}\right|$	0

*f*_12_	[−100, 100]^*D*^	30	Schwefel's Problem 2.21	*f*(*x*) = max_*i*_{|*x*_*i*_|, 1 ≤ *i* ≤ *n*}	0

*f*_13_	[−10, 10]^*D*^	30	Rosenbrock	*f*(*x*) = ∑_*i*=1_^*n*−1^‍[100(*x*_*i*+1_ − *x*_*i*_^2^)^2^ + (*x*_*i*_ − 1)^2^]	0

*f*_14_	[−100, 100]^*D*^	30	Step	*f*(*x*) = ∑_*i*=1_^*n*^‍(⌊*x*_*i*_ + 0.5⌋)^2^	0

*f*_15_	[−1.28, 1.28]^*D*^	30	Quartic (noise)	*f*(*x*) = ∑_*i*=1_^*n*^‍*ix*_*i*_^4^ + random[0,1)	0

*f*_16_	[−500, 500]^*D*^	30	Schwefel	$f(x)=-\sum_{i=1}^{n}‍x_{i}\sin\left(\sqrt{\left|x_{i}\right|}\right)$	− 12569.5

*f*_17_	[−5.12, 5.12]^*D*^	30	Rastrigin	*f*(*x*) = [*x*_*i*_^2^ − 10cos(2π*x*_*i*_) + 10]	0

*f*_18_	[−5, 10] × [0, 15]	2	Branin	$f(x)={\left(x_{2}-\frac{5.1}{4\pi^{2}}x_{1}^{2}+\frac{5}{\pi }x_{1}-6\right)}^{2}+10\left(1-\frac{1}{8\pi }\right)\cos x_{1}+10$	0.398

*f*_19_	[−2, 2]^*D*^	2	Goldstein-Price	fx=1+(x1+x2+1)219-14x1+3x12-14x2+6x1x2+3x22fx=× 30+2x1-3x2218-32x1+12x12+48x2-36x1x2+27x22	3

*f*_20_	[0, 1]^*D*^	4	Hartman's Family (*n* = 3)	*f*(*x*) = −∑_*i*=1_^4^‍*c*_*i*_exp⁡[∑_*j*=1_^3^‍*a*_*ij*_(*x*_*j*_ − *p*_*ij*_)^2^]	− 3.86

*f*_21_	[0, 1]^*D*^	6	Hartman's Family (*n* = 6)	*f*(*x*) = −∑_*i*=1_^4^‍*c*_*i*_exp⁡[∑_*j*=1_^6^‍*a*_*ij*_(*x*_*j*_ − *p*_*ij*_)^2^]	− 3.32

*f*_22_	[0, 10]^*D*^	4	Shekel's Family (*m* = 5)	*f*(*x*) = −∑_*i*=1_^5^‍[(*x* − *a*_*i*_)(*x* − *a*_*i*_)^*T*^ + *c*_*i*_]^−1^	− 10

*f*_23_	[0, 10]^*D*^	4	Shekel's Family (*m* = 7)	*f*(*x*) = −∑_*i*=1_^7^‍[(*x* − *a*_*i*_)(*x* − *a*_*i*_)^*T*^ + *c*_*i*_]^−1^	− 10

*f*_24_	[0, 10]^*D*^	4	Shekel's Family (*m* = 10)	*f*(*x*) = −∑_*i*=1_^10^‍[(*x* − *a*_*i*_)(*x* − *a*_*i*_)^*T*^ + *c*_*i*_]^−1^	− 10

*f*_25_	[−65.536, 65.536]^*D*^	2	Shekel's Foxholes	$f(x)={\left[\frac{1}{500}+\sum_{j=1}^{25}‍\frac{1}{j+\sum_{i=1}^{2}‍\left(x_{i}-a_{ij}\right){}^{6}}\right]}^{-1}$	1

*f*_26_	[−5, 5]^*D*^	4	Kowalik	$f(x)=\sum_{i=1}^{11}‍{\left[a_{i}-\frac{x_{1}\left(b_{i}^{2}+b_{i}x_{2}\right)}{b_{i}^{2}+b_{i}x_{3}+x_{4}}\right]}^{2}$	0.0003075

## Appendices

### A. Unconstrained Global Optimization Problems

See Tables 8 and 9.

### B. Set of Constrained Global Optimization Problems Used in Our Experiments

#### B.1. CP1

Consider the following:

(B.1) Min⁡   f(x)=5∑d=14xd−5∑d=14xd2−∑d=513xd,subject  to   g1(x)=2x1+2x2+x10+x11−10≤0,hhhhhhh hg2(x)=2x1+2x3+x10+x12−10≤0,hhhh hhhhg3(x)=2x2+2x3+x11+x12−10≤0,hhhhhh hhg4(x)=−8x1+x10≤0,hhhh hhhhg5(x)=−8x2+x11≤0,hhhhh hhhg6(x)=−8x3+x12≤0,hhhhhh hhg7(x)=−2x4−x5+x10≤0,hhhhhh hhg8(x)=−2x6−x7+x11≤0,hhhhhh hhg9(x)=−2x8−x9+x12≤0,

where bounds are 0 ≤ *x*_*i*_ ≤ 1  (*i* = 1,…, 9,13), 0 ≤ *x*_*i*_ ≤ 100  (*i* = 10,11,12). The global optimum is at *x* ^*^ = (1,1, 1,1, 1,1, 1,1, 1, , 3,3, 3,1), *f*(*x* ^*^) = −15.

#### B.2. CP2

Consider the following:

(B.2) Min⁡   f(x)=5.3578547x2+0.8356891x1x5hhhhhhhhhhhihihh+37.293239x1−40792.141,subject  to   g1(x)=85.334407+0.0056858x2x5hhhhhhhhhhhhhhhh+0.0006262x1x4−0.0022053x3x5hhhhhhhhhhhhhhhh−92≤0,hhhhhhh hg2(x)=−85.334407−0.0056858x2x5hhhhhhhhhhhhhhhh−0.0006262x1x4+0.0022053x3x5hhhhhhhhhhhhhh≤0,hhhhhhh hg3(x)=80.51249+0.0071317x2x5hhhhhhhhhhhhhhhh+0.0029955x1x2−0.0021813x2hhhhhhhhhhhhhhhh−110≤0,hhhhhhh hg4(x)=−80.51249−0.0071317x2x5hhhhhhhhhhhhhhhh+0.0029955x1x2−0.0021813x2hhhhhhhhhhhhhhhh+90≤0,hhhhhhh hg5(x)=9.300961−0.0047026x3x5hhhhhhhhhhhhhhhh−0.0012547x1x3−0.0019085x3x4hhhhhhhhhhhhhhhh−25≤0,hhhhhhh hg6(x)=−9.300961−0.0047026x3x5hhhhhhhhhhhhhhhh−0.0012547x1x3−0.0019085x3x4hhhhhhhhhhhhhhhh+20≤0,

where 78 ≤ *x*_1_ ≤ 102, 33 ≤ *x*_2_ ≤ 45, and 27 ≤ *x*_*i*_ ≤ 45  (*i* = 3,4, 5). The optimum solution is *x* ^*^ = (78,33,29.995256025682,45,36.775812905788), where *f*(*x* ^*^) = −30665.539. Constraints *g*_1_ and *g*_6_ are active.

#### B.3. CP3

Consider the following:

(B.3) Min⁡   f(x)=x1−103+x2−203,subject  to   g1(x)=−x1−52−x2−52+100≤0,hhhhhhh hg2(x)=x1−62+x2−52−82.81≤0,

where 13 ≤ *x*_1_ ≤ 100 and 0 ≤ *x*_2_ ≤ 100. The optimum solution is *x* ^*^ = (14.095,0.84296) where *f*(*x* ^*^) = −6961.81388. Both constraints are active.

#### B.4. CP4

Consider the following:

(B.4) Min⁡   fx=x12+x22+x1x2−14x1−16x2hhhhhhhhhihhh h+x3−102+4x4−52+x5−32hhhhhhhhhihhh h+2x6−12+5x72+7x8−112hhhhhhhhhihhh h+2x9−102+x10−72+45,subject  to   g1x=−105+4x1+5x2−3x7+9x8≤0,hhhhhhh hg2x=10x1−8x2−17x7+2x8≤0,hhhhhhh hg3x=−8x1+2x2+5x9−2x10−12≤0,hhhhhhh hg4x=3x1−22+4x2−32+2x32hhhhhhhhhhhhhhhh−7x4−120≤0,hhhhhhh hg5x=5x12+8x2+x3−62−2x4hhhhhhhhhhhhhhhh−40≤0,hhhhhhh hg6x=x12+2x2−22−2x1x2+14x5hhhhhhhhhhhhhhhh−6x6≤0,hhhhhhh hg7x=0.5x1−82+2x2−42+3x52hhhhhhhhhhhhhhhh−x6−30≤0,hhhhhhh hg8x=−3x1+6x2+12x9−82−7x10≤0,

where −10 ≤ *x*_*i*_ ≤ 10  (*i* = 1,…, 10). The global optimum is *x* ^*^ = (2.171996, 2.363683, 8.773926, 5.095984, 0.9906548, 1.430574, 1.321644, 9.828726, 8.280092,8.375927), where *f*(*x* ^*^) = 24.3062091. Constraints *g*_1_, *g*_2_, *g*_3_, *g*_4_, *g*_5_, and *g*_6_ are active.

#### B.5. CP5

Consider the following:

(B.5) Min⁡   f(x)=x12+x2−12,subject  to   g1(x)=x2−x12=0,

where 1 ≤ *x*_1_ ≤ 1, 1 ≤ *x*_2_ ≤ 1. The optimum solution is $x^{*}=(\pm 1/\sqrt{\left(2\right)},1/2)$, where *f*(*x* ^*^) = 0.7499.

#### B.6. Welded Beam Design Optimisation

The welded beam design is a standard test problem for constrained design optimisation [55, 56]. There are four design variables: the width *w* and length *L* of the welded area and the depth *d* and thickness *h* of the main beam. The objective is to minimise the overall fabrication cost, under the appropriate constraints of shear stress *τ*, bending stress *σ*, buckling load *P*, and maximum end deflection *δ*. The optimization model is summarized as follows, where *x* ^*T*^ = (*w*, *L*, *d*, *h*):

(B.6) Minimise f(x)=1.10471w2L+0.04811dh(14.0+L),subject  to    g1(x)=w−h≤0,hhhhhhhhhg2(x)=δ(x)−0.25≤0,hhhhhhhhhg3(x)=τ(x)−13,600≤0,hhhhhhhhhg4(x)=σ(x)−30,000≤0,hhhhhhhhhg5(x)=1.10471w2+0.04811dh(14.0+L)hhhhhhhhhhhhhhh−5.0≤0,hhhhhhhhhg6(x)=0.125−w≤0,hhhhhhhhhg7(x)=6000−P(x)≤0,

where

(B.7) $$\begin{array}{c} \sigma \left(x\right)=\frac{504,000}{hd^{2}},\;\;D=\frac{1}{2}\sqrt{L^{2}+{\left(w+d\right)}^{2}},\\ Q=6000\left(14+\frac{L}{2}\right),\;\;\delta =\frac{65,856}{30,000hd^{3}},\\ J=\sqrt{2}wL\left(\frac{L^{2}}{6}+\frac{{\left(w+d\right)}^{2}}{2}\right),\;\;\alpha =\frac{6000}{\sqrt{2}wL},\\ \beta =\frac{QD}{J},\;\;P=0.61423\times 10^{6}\frac{dh^{3}}{6}\left(1-\frac{\sqrt[{d}]{30/48}}{28}\right),\\ \tau \left(x\right)=\sqrt{\alpha^{2}+\frac{\alpha \beta L}{D}+\beta^{2}}. \end{array}$$

#### B.7. Speed Reducer Design Optimization

The problem of designing a speed reducer [57] is a standard test problem. It consists of the design variables as face width *x*_1_, module of teeth *x*_2_, number of teeth on pinion *x*_3_, length of the first shaft between bearings *x*_4_, length of the second shaft between bearings *x*_5_, diameter of the first shaft *x*_6_, and diameter of the first shaft *x*_7_ (all variables are continuous except *x*_3_ that is integer). The weight of the speed reducer is to be minimized subject to constraints on bending stress of the gear teeth, surface stress, transverse deflections of the shafts, and stresses in the shaft [55]. The mathematical formulation of the problem, where *x* ^*T*^ = (*x*_1_, *x*_2_, *x*_3_, *x*_4_, *x*_5_, *x*_6_, *x*_7_), is as follows:

(B.8) Minimise fx=0.7854x1x22hhhhhhhhhhhhhhh·(3.3333x32+14.9334x343.0934)hhhhhhhhhhhhhhh−1.508x1(x62+x73)hhhhhhhhhhhhhhh+7.4777(x63+x73)hhhhhhhhhhhhhhh+0.7854(x4x62+x5x72),subject  to g1(x)=27x1x22x3−1≤0,hhhhhhhhhg2(x)=397.5x1x22x32−1≤0,hhhhhhhhhg3(x)=1.93x43x2x3x64−1≤0,hhhhhhhhhg4(x)=1.93x53x2x3x74−1≤0,hhhhhhhhhg5(x)=1.0110x63745.0x4x2x32+16.9×106hhhhhhhhhhhhhhhi−1≤0,hhhhhhhhhg6(x)=1.085x73745.0x5x2x32+157.5×106hhhhhhhhhhhhhhhi−1≤0,hhhhhhhhhg7(x)=x2x340−1≤0,hhhhhhhhhg8(x)=5x2x1−1≤0,hhhhhhhhhg9(x)=x112x2−1≤0,hhhhhhhhhg10(x)=1.5x6+1.9x4−1≤0,hhhhhhhhhg11(x)=1.1x7+1.9x5−1≤0.

The simple limits on the design variables are 2.6 ≤ *x*_1_ ≤ 3.6, 0.7 ≤ *x*_2_ ≤ 0.8, 17 ≤ *x*_3_ ≤ 28, 7.3 ≤ *x*_4_ ≤ 8.3, 7.8 ≤ *x*_5_ ≤ 8.3, 2.9 ≤ *x*_6_ ≤ 3.9, and 5.0 ≤ *x*_7_ ≤ 5.5.

#### B.8. Spring Design Optimisation

The main objective of this problem [58, 59] is to minimize the weight of a tension/compression string, subject to constraints of minimum deflection, shear stress, surge frequency, and limits on outside diameter and on design variables. There are three design variables: the wire diameter *x*_1_, the mean coil diameter *x*_2_, and the number of active coils *x*_3_ [55]. The mathematical formulation of this problem, where *x* ^*T*^ = (*x*_1_, *x*_2_, *x*_3_), is as follows:

(B.9) Minimize f(x)=(x3+2)x2x12,subject  to g1(x)=1−x23x37,178x14≤0,hhhhihhhhhg2(x)=4x22−x1x212,566x2x13−x14+15,108x12hhhhhhhhhhhhhhhh−1≤0,hhhhhihhhhg3(x)=1−140.45x1x22x3≤0,hhhhhihhhhg4(x)=x2+x11.5−1≤0.

The simple limits on the design variables are 0.05 ≤ *x*_1_ ≤ 2.0, 0.25 ≤ *x*_2_ ≤ 1.3, and 2.0 ≤ *x*_3_ ≤ 15.0.
